# Two-minute walk distance reference equations for middle-aged and elderly Chinese individuals with obesity

**DOI:** 10.1371/journal.pone.0273550

**Published:** 2022-08-24

**Authors:** Jia Zhang, Yingying Zou, Zibin Wang, Xiaoshu Chen, Jingye Pan, Haizhu Yu, Enci Li, He Zou

**Affiliations:** 1 Department of Medical Inspection, Wenzhou People’s Hospital, The Wenzhou Third Clinical Institute Affiliated with Wenzhou Medical University, Wenzhou, Zhejiang, China; 2 School of Laboratory Medicine and Life Sciences, Wenzhou Medical University, Wenzhou, Zhejiang, China; 3 Digestive System Department, The Third Affiliated Hospital of Qiqihar Medical College, Qiqihar, Heilongjiang, China; 4 Obstetrics Department, Wenzhou People’s Hospital, The Wenzhou Third Clinical Institute Affiliated with Wenzhou Medical University, Wenzhou, Zhejiang, China; 5 Department of Cardiovascular Medicine, Wenzhou People’s Hospital, The Wenzhou Third Clinical Institute Affiliated with Wenzhou Medical University, Wenzhou, Zhejiang, China; 6 Department of General and Intensive Medical Care, The First Affiliated Hospital of Wenzhou Medical University, Wenzhou, Zhejiang, China; 7 Department of General Practice, Zhejiang Hospital, Hangzhou, Zhejiang, China; 8 Nursing Department, The First Affiliated Hospital of Wenzhou Medical University, Wenzhou, Zhejiang, China; PLOS ONE, UNITED KINGDOM

## Abstract

**Background and objective:**

While the six-minute walk test (6MWT) is often used to assess exercise capacity, the less well-known two-minute walk test (2MWT) is more feasible for some patients. In previous studies, we developed reference equations for the two-minute walk distance (2MWD) for healthy Chinese adults. However, our study did not recruit people with obesity, and the reference equations did not apply to participants with a body mass index (BMI) > 30 kg/m^2^. The main objective of this study was to establish reference equations for the 2MWD among middle-aged and elderly Chinese individuals with obesity.

**Methods:**

A total of 295 individuals were recruited. The participants underwent two 2MWTs, with the longer of the two 2MWDs used for further analyses. The reference equations for the 2MWD were developed using stepwise multiple regression analysis. The newly established equations for the 2MWD were then compared with the existing equations.

**Results:**

The mean 2MWD of the participants was 176±20 m. Age and BMI were identified as independent factors that influenced the 2MWD and explained 28% and 32% of the variance in walking distance for the male and female groups, respectively. The reference equations for the 2MWD were as follows:

$$\begin{array}{l} Male:2MWD(m)=443.058-[age(yr)x6.782]+[BMI(\mathrm{kg}/m^{2})x0.847];r^{2}=0.283\\ Female:2MWD(m)=300.160-[age(yr)x0.841]-[BMI(\mathrm{kg}/m^{2})x2.541];r^{2}=0.315 \end{array}$$

**Conclusion:**

This study resulted in the development of reference equations for predicting 2MWD among middle-aged and elderly Chinese people with obesity. These equations will be a clinically valuable tool for evaluating functional capacity, determining prognoses and monitoring treatment in middle-aged and elderly Chinese people with obesity.

## Introduction

The six-minute walk test (6MWT) is a relatively simple, safe and inexpensive way to evaluate functional capacity and was recommended by the American Thoracic Society in 2002 [1]. The 6MWT has been used to assess functional capacity, determine prognosis and monitor the treatment of various subjects [2–4]. Nevertheless, the use of an alternative, the two-minute walk test (2MWT) is actually reasonable in some settings and populations. The 2MWT takes less time than the 6MWT, and two minutes is rarely exceeded during incidental bouts of walking among some patients with special diseases [5]. In addition, there is often a strong correlation between the two-minute walk distance (2MWD) and the six-minute walk distance (6MWD) [6]. Some patients who have terminal heart disease or are hospitalized for severe chronic obstructive lung disease often “give up” before walking for six minutes and are usually only willing to walk for approximately two minutes [6].

In previous studies, we established reference equations for the 2MWD of healthy Chinese adults [7]. However, our study did not recruit people with obesity, and the reference equations were not applicable to individuals with body mass index (BMI) > 30 kg/m^2^. Several studies [8] have shown that reference equations for walking distance derived primarily from healthy, normal-weight people are not suitable for individuals with obesity, and specific reference equations for walking distance for individuals with obesity should be established.

Thus, the objectives of the present study were 1) to analyse the anthropometric variables and walking distance comparing sex and age; 2) to set up reference equations for the 2MWD among middle-aged and elderly Chinese people with obesity; and 3) to compare the 2MWD equations obtained in this study with existing equations for individuals in the same age range.

## Methods

### Participants

We collected data over a 30-month period from May 2019 to December 2020, and individuals (BMI > 30 kg/m^2^) older than 40 years with obesity were recruited from three local communities. The purpose of this study was explained to potential participants prior to recruitment. The participants were required to complete a health questionnaire before participating in the test, and the researchers needed to verify the results of the questionnaires. We obtained the participants’ written informed consent before the study began. This study was approved by the Ethics Committee of Wenzhou People’s Hospital.

Individuals who met the following criteria were excluded from the study:

- age > 75 years;
- self-reported disease symptoms (including heart disease, lung disease and other organ or blood neurological diseases);
- baseline heart rate (HR) < 50 bpm or ≥ 100 bpm;
- baseline systolic blood pressure (SBP) ≥ 180 mmHg or diastolic blood pressure (DBP) ≥ 100 mmHg;
- trouble walking or need for a hearing aid.

### Physical examination

The participants underwent anthropometric measurements, which were performed by skilled operators following the procedures described in the anthropometric standardization reference manual. Age was verified by each subject’s identity card. Height was measured to the nearest 0.1 cm with a height gauge; for this measurement, the participants needed to take off their shoes and stand with their back straight. An electronic scale was used to measure body weight (kg) to the nearest 0.1 kg, and the BMI algorithm = weight/height^2^ (represented by kg/m^2^) was used to calculate and obtain the BMI of each subject. The percentage of the predicted maximum heart rate (%) = heart rate after 2MWT/(220—age).

### Two-minute walk test

The 2MWT is a revised version of the 6MWT developed from ATS guidelines in 2002 [1]. The 2MWTs were conducted in an enclosed corridor that was 30 metres long, wide and tall. The participants were asked to rest on nearby chairs for at least 10 minutes. Researchers measured and recorded some data before the study, such as heart rate, oxygen saturation and blood pressure. Blood pressure was calculated according to the National Institutes of Health (NIH) guidelines [9]. Then, the participants were asked to walk as fast as possible in the court for 2 minutes, but they were not allowed to run. If there were severe symptoms of exercise intolerance, the participants could stop and rest or slow down their speed. We would encourage them to continue walking again as soon as possible after they recovered. The researchers told the participants how long it would be before the 2MWT ended while giving them encouragement such as “very good” and “one minute left, thirty seconds left.” The distances that the participants walked for two minutes were recorded as the 2MWDs. The researchers measured two 2MWDs with an interval of 2 hours for every participant, and the longer 2MWD was used for further analysis.

### Methods

After an in-depth analysis of demographic characteristics, the main variables in this study mainly had a normal distribution, and Kolmogorov-Smirnov tests were used to measure them. The measured data are expressed as the means ± standard deviations (SDs) or as numbers and percentages, as appropriate. Descriptive analysis was used to evaluate the characteristics of the participants. Independent Student’s *t test*s were used to analyse the relationship between the 2MWD and the characteristics of categorical variables. In this study, we used paired sample *t tests* to compare the measured 2MWDs of our participants and the predicted 2MWDs based on previously published reference equations derived from other studies [7,10–12]. According to Bland-Altman analysis and calculation of the intraclass correlation coefficient (ICC), the repeatability of the two 2MWDs was calculated [13]. First, the correlations between the 2MWD and variables (i.e., age, height, weight and BMI) were evaluated by Pearson analysis of an individual variable. Second, the reference equations of the 2MWD were established by forward stepwise multivariate regression analysis. In each procedure, the most significant categorical variable was added to the model, and then the same procedure was followed until no additional significant variables remained. A p value > 0.05 was used to detect whether the variable was entered or deleted. Data were analysed using SPSS for Windows statistical software (version 20.0; SPSS, Inc., Chicago, IL). A p value < 0.05 was considered significant in all analyses.

## Results and discussion

### Demographic characteristics and 2MWT results

**Fig 1:**
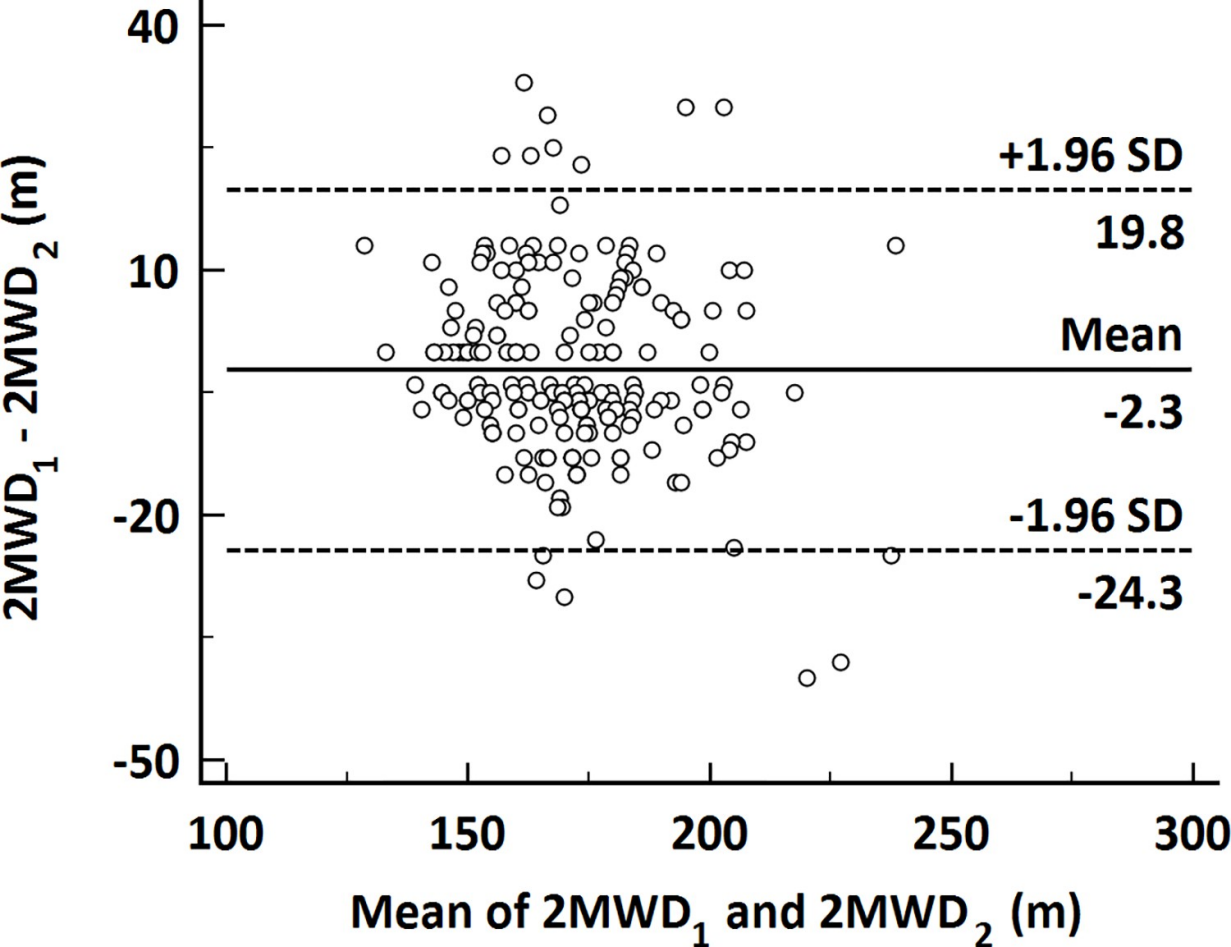
Bland-Altman plot of the results for performance in the first and second 2MWTs.

Two hundred ninety-five healthy individuals were recruited for this study. Eighty-one individuals were excluded from this study (foot sprain: n = 2; cardiac disease: n = 26; abnormal basal heart rate: n = 10; unstable hypertension: n = 30; pulmonary disease: n = 6 and cerebral disease: n = 7). Finally, 214 individuals (102 men and 112 women) completed the 2MWTs, with no participants prematurely requiring rest during the test or terminating the test. Table 1 shows the characteristics of these participants and the 2MWT results. Males were significantly taller and heavier than females in our study, and there was a difference in BMI between the sexes. The mean 2MWD for all the participants was 176±20 m. The mean distance was 182 ± 21 m for males and 171 ± 16 m for females, and the difference was significant (p < 0.001). Age- and sex-stratified values of the 2MWD are summarized in Table 2. The mean 2MWDs of the first and second test sessions were 173 ± 20 m and 176 ± 20 m, respectively. The Bland-Altman chart (Fig 1) shows the mean difference between the first and second 2MWDs, and the reliability of the two 2MWTs was good (ICC = 0.89). We were able to see a difference in heart rate between the sexes. By the end of the test, participants’ heart rates had reached our predicted maximum of approximately 65%.

**Table 1:** Characteristics and 2MWT results of the study participants.

Characteristic	Males (n = 102)	Females (n = 112)	p value*	Total (n = 214)
Age, years	56.9±10.23	57.9±10.22	NS	57.5±10.21
Height, cm	166.9±6.29	154.4±5.40	<0.001	160.4±8.57
Weight, kg	87.4±7.26	76.0±6.87	<0.001	81.4±9.04
BMI, kg/m^2^	31.3±1.28	31.9±1.75	<0.05	31.6±1.57
HR_1_, bpm	74.3±8.99	71.8±8.53	<0.05	73.0±8.82
HR_2_, bpm	106.4±11.87	104.4±11.53	NS	105.4±11.71
Borg	2.1±0.70	2.2±0.37	NS	2.1±0.57
2MWD, m	182.4±21.10	170.5±16.36	<0.001	176.2±19.66

Values are expressed as the mean±SD. *p value between males and females. 2MWT: Two-minute walk test. HR_1_: Basic heart rate. HR_2_: Heart rate after 2MWT. 2MWD: Two-minute walk distance.

**Table 2:** Age- and gender- stratified values of the 2MWD (m).

Age, years (n)	Males (n = 102)	Females (n = 112)	p value*	Total (n = 214)
40–49 (n = 62)	187.9±21.39	177.3±13.60	<0.05	182.8±18.68
50–59 (n = 63)	186.3±21.23	176.4±14.77	<0.05	181.3±18.76
60–69 (n = 56)	181.0±18.59	169.6±15.45	<0.05	174.9±17.77
70–75 (n = 33)	162.6±13.00	151.9±7.88	<0.05	156.1±11.35

Values are expressed as the mean±SD. *p value between males and females. 2MWD: Two-minute walk distance.

### Reference equations for the 2MWD

Table 3 shows the summary of the relationships between the 2MWD and the variables characteristics of the male and female participants. According to the univariate linear regression analysis, as expected, the variables (age, height and BMI) were correlated with the 2MWD. Therefore, the variables (age, height and BMI) were included in the stepwise multivariate regression analysis. Age and BMI were identified as independent factors that influenced the 2MWD and explained 28% and 32% of the variance in distance for the male and female groups, respectively (Table 4).

**Table 3:** Pearson correlations between the variables and the 2MWD (m).

Variable	Males (n = 102)		Females (n = 112)	
	**r value**	**p-value**	**r value**	**p-value**
Age	-0.359	<0.001	-0.504	<0.001
Height	0.170	<0.05	0.173	<0.05
Weight	-0.027	NS	-0.004	NS
BMI	-0.362	<0.001	-0.231	<0.05

2MWD: Two-minute walk distance; r value: Pearson’s correlation coefficient; BMI: Body mass index.

**Table 4:** Results of stepwise multiple linear regression analysis of the independent variables that explained the 2MWD (m).

	Males			Females		
	B	SE	p-value	B	SE	p-value
Constant	443.058	46.072	<0.001	300.160	25.133	<0.001
Age	-6.782	1.396	<0.001	-0.841	0.126	<0.001
BMI	-0.847	0.175	<0.001	-2.541	0.736	0.001
R^2^	0.297			0.327		
Change in R^2^	0.283			0.315		

B: Uunstandardized coefficients. SE: Standard error.

The reference equations for the 2MWD were as follows: $\begin{array}{l} Male:2MWD(m)=443.058-[age(yr)x6.782]+[BMI(\mathrm{kg}/m^{2})x0.847];r^{2}=0.283\\ Female:2MWD(m)=300.160-[age(yr)x0.841]-[BMI(\mathrm{kg}/m^{2})x2.541];r^{2}=0.315. \end{array}$

Table 5 shows a comparison between the measured 2MWDs of the participants and the predicted 2MWDs by the previous reference equations [7,10–12] for the same age ranges. The reference equations from Selman et al. [12], Hill et al. [11] and Zhang et al. [7] overestimated the walking distances of our participants, and the reference equations by Bohannon et al. [10] underestimated the distances. The differences between the measured 2MWDs and the predicted 2MWDs for the same age range based on the equations reported in the studies by Selman et al. [12], Bohannon et al. [10], Mirza et al. [11] and Zhang et al. [7] were -22.3±23.27 m, -19.0±18.02 m, 7.0±16.53 and -12.3±18.41, respectively.

**Table 5:** Measured 2MWDs (m) and predicted 2MWDs (m) for the same age range based on the equations reported in previous studies.

Study	Measured (m)	Predicted (m)	Measured-predicted (m)
Mirza et al	176.2±19.66	198.4±25.59	-22.3±23.27*
Selman et al.	176.2±19.66	195.2±15.91	-19.0±18.02*
Bohannon et al.	176.2±19.66	169.2±12.04	7.0±16.53*
Zhang et al	176.2±19.66	188.4±15.74	-12.3±18.41*

*p<0.05 according to Student’s *t test*. 2MWD: Two-minute walk distance.

## Discussion

This study analysed the anthropometric variables and 2MWDs of middle-aged and elderly Chinese individuals with obesity, established reference equations for their 2MWDs, and compared the current 2MWD equations with previous equations. The stepwise multivariate regression analysis showed that age and BMI were independently predictive of 2MWD among the participants of both sexes. To our knowledge, this is the first study that incorporates anthropometric parameters in the prediction of 2MWDs of middle-aged and elderly Chinese people of both sexes with obesity.

**Fig 2:**
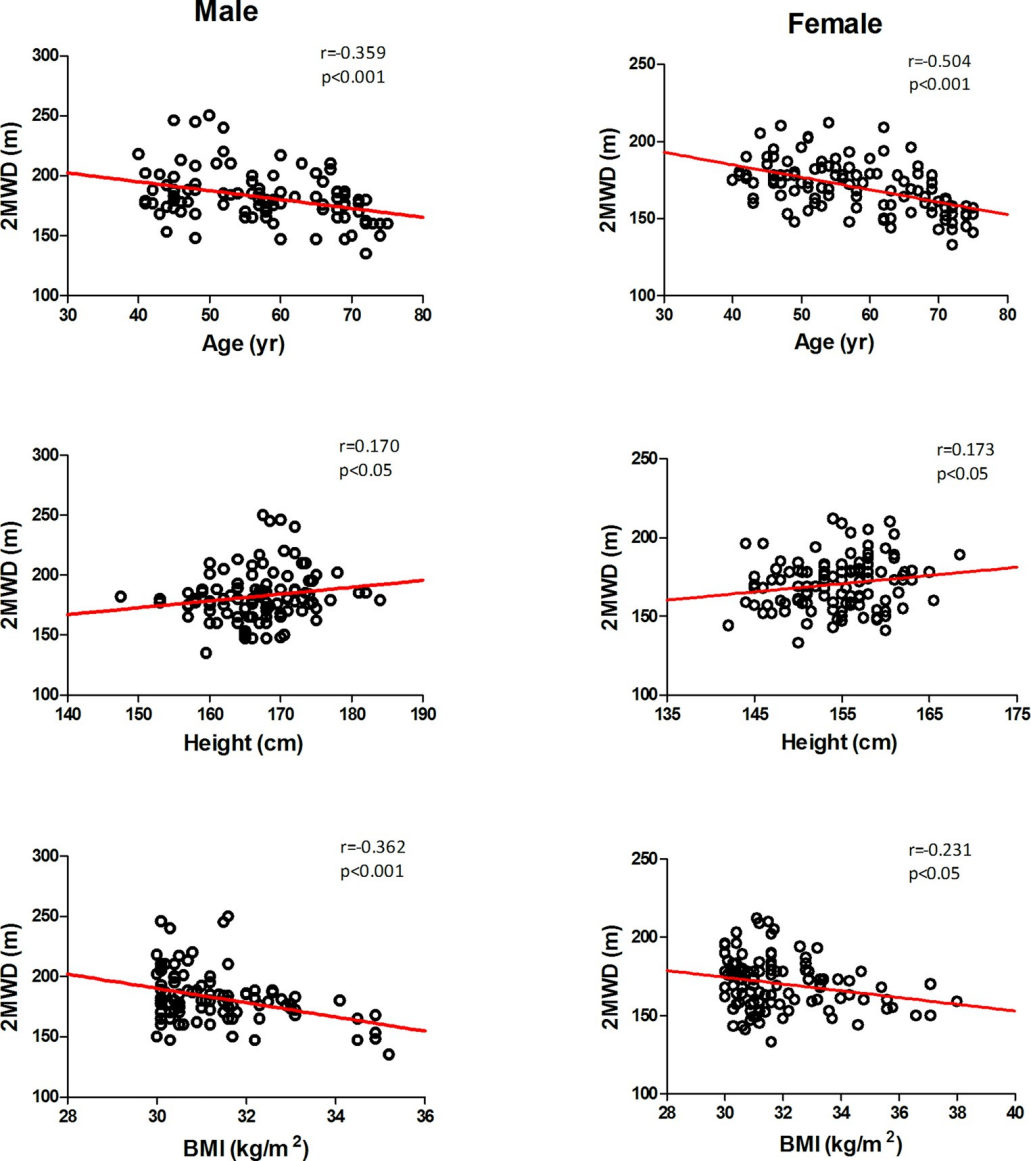
The relationship between 2MWD and age, height and BMI for females and males.

Age and BMI were negatively correlated with the 2MWD in our study (Fig 2). This is probably related to the loss of muscle mass as we age and the decrease in oxygen intake. Individuals with obesity experience certain complications with increases in BMI, which usually manifest as activity disorders caused by heart and breathing limitations [14,15]; weakened skeletal muscle strength is also one of the main causes of disability [16,17]. Other possible reasons include skin friction caused by fat deposition in the thighs, increased plantar pressure, and physical discomfort caused by exercise for people with a BMI that is higher than normal [18–21]. Height and walking distance were positively correlated in our study (Fig 2). This could be because, in general, taller individuals with larger strides cover more walking distance in the same amount of time than others. However, our study focused on people with obesity, and BMI was more meaningful than height for the 2MWD, so height was not included in the final regression equation. From this study, we observed that the resting heart rate of male subjects was lower than that of female subjects. Previous studies have shown that the difference in heart rate is correlated with sex, and the resting heart rate of men is lower than that of women [22]. This may be because men and women have different abilities to regulate the baroreflex heart rate, and oestrogen contributes to the ability to regulate the baroreflex heart rate in people [23]. There was a significant difference in the walking distance samples for men and women. Men tend to walk longer distances than women, probably due to their higher muscle mass and greater athletic ability.

By measuring walking distance performance twice, we can see that the mean 2MWD during the second test period was greater than the mean 2MWD during the first test period. This finding is the same as previous findings for the 2MWT [4,24,25]. The reason why the distance can be increased may be related to the learning effect. Participants may have overcome negative emotions, improved coordination, and adopted an appropriate stride distance in the second 2MWT. Although the walking distance in the second 2MWT was greater than that in the first test in our study, the reliability of the two 2MWTs was good (ICC = 0.83). Previous studies have also proven that two 2MWTs are reliable [7,10,11,26], which is consistent with the current study. Fourteen participants had error values outside the 95% confidence interval (Fig 1). Seven participants showed an increase in 2MWD in the second test, which might be due to familiarity with the 2MWT. Eight participants showed shorter walking distances in the second 2MWT, which might be due to greater fatigue during the second test due to better performance in the first 2MWT.

Among anthropometric parameters, age and BMI showed the highest correlation with 2MWD. According to the stepwise multivariate regression analysis, age and BMI were independently predictive of 2MWD among subjects of both sexes with obesity. After applying mathematical analysis, such as the theory proposed by Hulens et al. [27], we found that other factors that correlate with walking distance in 2 minutes would certainly benefit the prediction equations. In addition to anthropometric parameters, other factors (heart rate, blood pressure, muscle strength and lifestyle factors) also play an important role in predicting 2MWD, but they exist only in the equations and are impractical for clinical use.

The reference equations reported in previous studies could not accurately predict the 2MWD of our participants, and there was a significant difference between the measured 2MWD and predicted 2MWD for the same age range (p<0.05). These differences were often due to the test protocol, anthropometric factors, ethnic background and demographic differences among the participants. The reference equations by Bohannon et al. [10] underestimated the 2MWDs of our participants. Compared with individuals in our study, most of the participants in the study by Bohannon et al. [10] were overweight and obese, and they were taller; in addition, the test protocols were not all the same. For instance, the corridor was 15.2 m, encouragement was provided every 60 seconds during the 2MWT, and only a few participants had two 2MWTs in the study by Bohannon et al. [10]. In our study, we gave participants encouragement every 30 seconds during the 2MWT;. the corridor was 30 m, two 2MWTs were performed, and the greater 2MWD was used for further analysis. Some studies have shown that the frequency of encouragement during the test [28], the length of the corridor [29] and the use of a practice test [30] influence the walking distance. The reference equations by Selman et al. [12], Mirza et al. [11] and Zhang et al. [7] overestimated the 2MWDs of our participants. The studies by Selman et al. [12] and Zhang et al. [7] enrolled healthy, normal-weight subjects, and Mirza et al. [11] enrolled healthy individuals and only a low proportion of individuals with obesity. Although compared with our study, the studies by Selman et al. [12], Mirza et al. [11] and Zhang et al. [7] had a similar test protocol, the 2MWDs in these studies were greater than those in our sample, even at a lower workload. In addition to the daily physical activity of the participants, their mood and psychological factors may influence the 2MWDs [31].

There were some limitations in our study. First, although the sample size of our study was relatively large, a convenience sampling method was used. Second, we did not recruit individuals with obesity over 75 years of age. A large multicentre prospective study is needed to address these limitations.

## Conclusions

In summary, this study resulted in the development of reference equations for predicting the 2MWDs of middle-aged and elderly Chinese individuals with obesity. These equations will be a clinically valuable tool for evaluating functional capacity, determining prognoses and monitoring treatment in middle-aged and elderly Chinese individuals with obesity.

## Supporting information

S1 Dataset (XLSX)
